# Explainable Personality Prediction Using Answers to Open-Ended Interview Questions

**DOI:** 10.3389/fpsyg.2022.865841

**Published:** 2022-11-18

**Authors:** Yimeng Dai, Madhura Jayaratne, Buddhi Jayatilleke

**Affiliations:** Sapia&Co Pty Ltd., Melbourne, VIC, Australia

**Keywords:** personality prediction, HEXACO personality model, linguistic analysis, NLP, BERT

## Abstract

In this work, we demonstrate how textual content from answers to interview questions related to past behavior and situational judgement can be used to infer personality traits. We analyzed responses from over 58,000 job applicants who completed an online text-based interview that also included a personality questionnaire based on the HEXACO personality model to self-rate their personality. The inference model training utilizes a fine-tuned version of InterviewBERT, a pre-trained Bidirectional Encoder Representations from Transformers (BERT) model extended with a large interview answer corpus of over 3 million answers (over 330 million words). InterviewBERT is able to better contextualize interview responses based on the interview specific knowledge learnt from the answer corpus in addition to the general language knowledge already encoded in the initial pre-trained BERT. Further, the “Attention-based” learning approaches in InterviewBERT enable the development of explainable personality inference models that can address concerns of model explainability, a frequently raised issue when using machine learning models. We obtained an average correlation of *r* = 0.37 (*p* < 0.001) across the six HEXACO dimensions between the self-rated and the language-inferred trait scores with the highest correlation of *r* = 0.45 for Openness and the lowest of *r* = 0.28 for Agreeableness. We also show that the mean differences in inferred trait scores between male and female groups are similar to that reported by others using standard self-rated item inventories. Our results show the potential of using InterviewBERT to infer personality in an explainable manner using only the textual content of interview responses, making personality assessments more accessible and removing the subjective biases involved in human interviewer judgement of candidate personality.

## 1. Introduction

Understanding personality plays a critical role in making sense of one's own self and their relationships with others, especially within a work environment. To that end, personality is widely accepted as an indicator of job performance, job satisfaction, and tenure intention (Barrick and Mount, 1991; Salgado, 2002; Rothmann and Coetzer, 2003; Lounsbury et al., 2008, 2012; Ariyabuddhiphongs and Marican, 2015). The most common approach for assessing personality is to use a self-report personality questionnaire such as the NEO-PI-R (Costa and McCrae, 2008) or the HEXACO-PI-R (Lee and Ashton, 2018) that consists of a large number of personality related statements rated by the individual on a Likert scale. While decades of research have shown the validity and improved on the traditional approach of assessing personality (Morgeson et al., 2007a,b; Ones et al., 2007), adding a personality test to the recruitment process tends to increase the cost-to-hire and diminishes candidate experience since most personality tests are lengthy and tedious (Mcdaniel et al., 1994; Macan, 2009). Hence, personality assessments are not frequently included in hiring for most roles, especially in high-volume recruitment, despite its validity.

On the other hand, job interview remains the most common form of assessment in candidate selection and the ability to automatically infer personality from answers to job interview questions could replace lengthy personality assessments (Jayaratne and Jayatilleke, 2020). Moreover, a data-driven approach can help counter flaws in human judgement due to personal factors such as mood, own personality, and biases that unavoidably affect interview outcomes (Uleman, 1999; Ham and Vonk, 2003; Ma et al., 2011; Ferreira et al., 2012). When conducting a large number of interviews, human interviewers can hardly infer personality accurately and efficiently with clear explanations for each candidate. The ability to automate the inference of personality can offer more candidates the opportunity to express themselves and be heard, especially in high-volume recruitment where only a small fraction typically progress to a face-to-face interview.

**Figure 1:**
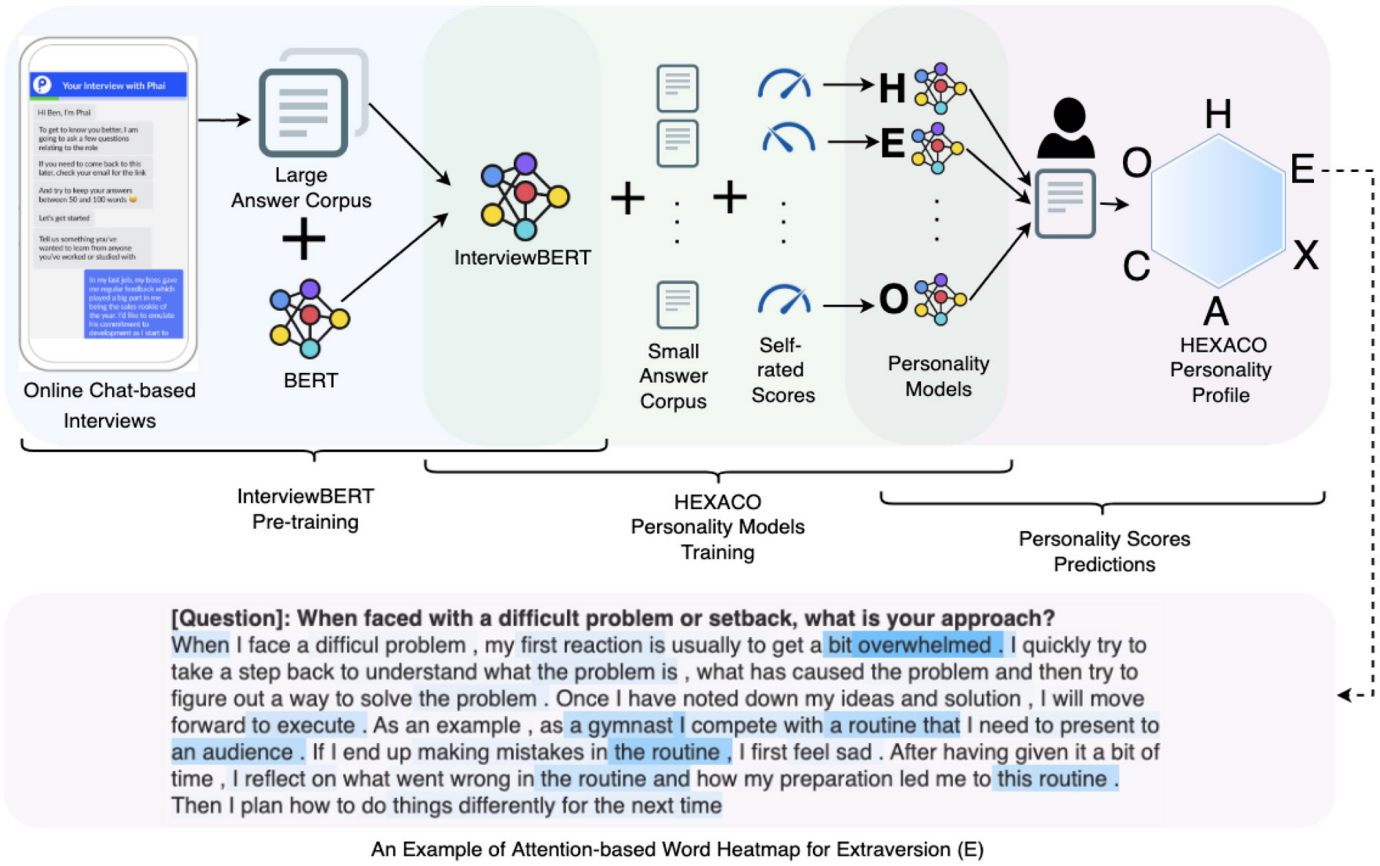
Illustration of InterviewBERT based HEXACO personality prediction.

In this work, we demonstrate how textual content from answers to interview questions related to past behavior and situational judgement can be used to infer personality traits reliably. Figure 1 shows the overview of our methodology. We used data from over 58,000 job applicants who completed an online chat interview that also included a personality questionnaire based on the six-factor HEXACO personality model (Ashton and Lee, 2007) to self-rate their personality. We proposed InterviewBERT, a variant of the state-of-the-art Bidirectional Encoder Representations from Transformers (BERT) (Devlin et al., 2019), which is a transformer (Vaswani et al., 2017) based machine learning technique for NLP pre-training. We extended BERT with a large interview answer corpus of over 3 million answers consisting of over 330 million words. InterviewBERT is able to better contextualize interview responses based on the interview specific knowledge learnt from the answer corpus in addition to the general language knowledge already encoded in the initial pre-trained BERT. We show the advantage of using context-specific answer representations to infer personality compared to context-free methods, and study different ways of using InterviewBERT to achieve context-specific answer representations. The use of InterviewBERT also differentiates our approach from previous work related to the inference of personality from interview responses such as Jayaratne and Jayatilleke (2020), that uses context-free NLP approaches.

Moreover, we show how the self-attention mechanism (Vaswani et al., 2017) in InterviewBERT can be used to develop more explainable personality inference models. Attention in this context is motivated by human behaviors seen in activities such as vision and reading comprehension where people pay varying levels of attention to different regions in an image or words in a text, according to different situations and goals. We use self-attention to capture the relationships between words and use the attention to provide a basis for providing explanations for personality inference outcomes.

Our results show the potential of algorithms to objectively infer a candidate's personality in an explainable manner using only the textual content of interview responses, presenting significant opportunities to remove the subjective biases involved in human interviewer judgement of candidate personality.

The main contributions of this paper are listed as follows.

1. We demonstrate that the textual content from answers to standard interview questions can be used to infer one's personality.
2. We propose the use of context-specific text representations for interview answers and propose InterviewBERT that extends the BERT model with a large interview response corpus.
3. We empirically investigate the performance of InterviewBERT based personality prediction using a real online interview dataset.
4. We show the language-level explainability of the InterviewBERT based prediction results.
5. We investigate the gender differences in personality traits inferred from interviews.

The rest of the paper is organized as follows. In Section 2, we provide a review of related work and introduce the details of our methodology preliminaries. In Section 3, we introduce the way we construct our data set (Section 3.1), present different methods of answer representations (Section 3.2), and outline the model to infer personality from answer representations (Section 3.3). In Section 4, we present the experimental results followed by a discussion in Section 5. In Section 6, we conclude with suggestions for future directions.

## 2. Background

In this section, we introduce the preliminaries of the HEXACO personality model used as the underlying personality model in our study (Section 2.1), and the related work around language and personality (Section 2.2). We also provide an overview of the methods we use to infer personality from textual content of interview responses. These include the different word and document representation approaches found in natural language processing (Section 2.3), the BERT model architecture and the self-attention mechanism (Section 2.4) that form the basis for the InterviewBERT model. We find that a lengthy discussion of the technical details of the above topics is out of the scope of this paper and refer the reader to the related work we reference under each topic.

### 2.1. HEXACO Model

HEXACO (Ashton and Lee, 2007) is a six-dimensional model of personality consisting of Honesty-humility (H), Emotionality (E), eXtraversion (X), Agreeableness (A), Conscientiousness (C), and Openness (O) as dimensions. Similar to the Big Five model (Goldberg, 1993) of personality, HEXACO model has its origins in lexical studies and subsequent factor analysis used to identify a minimal set of independent dimensions or personality traits and their underlying facets. It's relevant to note here that the use of lexical studies are grounded on the *lexical hypothesis* that claims descriptors of personality characteristics are encoded in language (Saucier and Goldberg, 1996), a fact we will re-visit in the next section. While there are similarities and subtle differences in the dimensions in HEXACO and the Big Five model, a key difference is the addition of the Honesty-Humility (H) dimension or the H-factor. The H-factor is especially important in the employment assessment context given it represents characteristics desired in a workplace environment such as modesty, fairness, and honesty. Previous studies have shown that the H-factor can help explain and predict workplace deviance (Pletzer et al., 2019), delinquency (Lee et al., 2005; de Vries and van Gelder, 2015), integrity (Lee et al., 2008), counterproductive work behavior and organizational citizenship (Anglim et al., 2018), and job performance (Johnson et al., 2011).

### 2.2. Language and Personality

Language analysis is a first-principles approach to understanding psychological constructs as studied in *psycholinguistics* and the application of *lexical hypothesis* in discovering personality dimensions. The field of psycholinguistics is dedicated to the study of the relationship between language and various psychological aspects related to language acquisition, understanding and human thought (Pinker, 2007; Gleitman and Papafragou, 2012). In Pinker (2007), the author details with extensive research on how we speak reveals what we think. More importantly personality models such as HEXACO and Big Five are grounded on the *lexical hypothesis*, which states that personality characteristics that are salient in people's daily transactions and relates to important social outcomes are encoded in language (John et al., 1988; Saucier and Goldberg, 1996). Advances in machine learning and natural language processing (NLP) have catalyzed the growing body of evidence showing the relationship between one's language use and personality (Boyd and Pennebaker, 2017). This relationship has been demonstrated in both informal contexts such as social media (Gill et al., 2009; Golbeck et al., 2011; Iacobelli et al., 2011; Park et al., 2015; Christian et al., 2021; Lucky and Suhartono, 2021) as well as in formal contexts such as self-narratives (Fast and Funder, 2008; Hirsh and Peterson, 2009), and job interviews (Jayaratne and Jayatilleke, 2020).

The language-personality relationship has been utilized to develop predictive machine learning models to accurately infer personality traits from blogs (Iacobelli et al., 2011), essays (Neuman and Cohen, 2014), microblogs (Twitter, Sina Weibo) (Golbeck et al., 2011; Sumner et al., 2012; Xue et al., 2017; Lucky and Suhartono, 2021), social media posts (Tadesse et al., 2018; Wang et al., 2019), etc. The success of such attempts has led researchers to propose computer generated personality predictions to “complement—and in some instances replace—traditional self-report measures, which suffer from well-known response biases and are difficult to scale” (Hall and Matz, 2020).

Language modeling within psychological sciences typically involves two types of approaches: the *closed-vocabulary* approach and the *open-vocabulary* approach. In closed-vocabulary approaches, words are assigned to psycho-socio-educational relevant categories to create dictionaries that are considered to represent that category. For example, words such as happiness, joy, etc. can be part of a dictionary for positive emotions. Linguistic Inquiry and Word Count (LIWC) (Pennebaker et al., 2015) is one such lexicon. Using the LIWC, researchers have found correlations among language patterns and personality (Fast and Funder, 2008; Gill et al., 2009; Hirsh and Peterson, 2009; Golbeck et al., 2011; Qiu et al., 2012). On the other hand, open-vocabulary approaches are more data-driven. In an open-vocabulary NLP system, algorithms process a large set of linguistic data and identify semantically related words through numerical word representation methods (We detail these methods in Section 2.3), which can be used to predict outcomes using supervised machine learning algorithms or gain further insights through exploration using unsupervised algorithms such as clustering. Compared to the closed-vocabulary methods, the open-vocabulary methods build upon the idea that words can be represented with numerical values based on how they co-occur, yielding to powerful language models that allow us to model words according to the contexts in which they appear rather than relying on assumptions about word-category relations. It eliminates the need for a human to have created categories and related dictionaries that limits the vocabulary known to learning algorithms. Open-vocabulary approaches are the current de facto standard for modeling language data and usually require a large amount of training data to learn the relationship between personality and language representation. Such predictive models have been demonstrated on textual data from social media with success (Schwartz et al., 2013; Park et al., 2015; Liu et al., 2016; Christian et al., 2021; Lucky and Suhartono, 2021).

### 2.3. Word and Document Representations

Natural language processing (NLP) requires representation of language and broadly two types of representations are used: context-free representations and context-specific representations. Traditional context-free representation methods include Bag of Words (BoW) and term frequency-inverse document frequency (TF-IDF) (Christopher et al., 2008) where BoW represents a document using the raw count of a term or n-gram (sequence of terms) in the corpus, while TF-IDF evaluates the importance of a term within a single document based on its occurrences across the document corpus. An obvious limitation of BoW and TF-IDF is that the meaning and term similarity are not encoded leaving unseen words as “out of vocabulary” when a trained model is applied on a new document. Further, they introduce very long and sparse input vectors, especially when the vocabulary is large. These context-free representations (in some instances along with other features) have been used in personality prediction from textual content (Iacobelli et al., 2011; Schwartz et al., 2013; Plank and Hovy, 2015; Verhoeven et al., 2016; Gjurković and Šnajder, 2018; Jayaratne and Jayatilleke, 2020). Neural word embedding methods such as Word2Vec (Mikolov et al., 2013) and GloVe (Pennington et al., 2014) attempt to address the capturing of contextual similarity of terms by providing a term level representation (called an embedding) by pre-training on a large corpus of documents (e.g., Wikipedia, open web crawl). For example, Word2Vec learns embeddings by predicting the current word based on its surrounding words or predicting the surrounding words given a current word (Skip-Gram). GloVe uses a count-based model, which learns embeddings by looking at how often a word appears in the context of another word within the corpus, focusing on the co-occurrence probabilities of words within a large training corpus of documents such as Wikipedia. Studies of personality inferences that use neural word embeddings include (Kamijo et al., 2016; Arnoux et al., 2017; Majumder et al., 2017; Jayaratne and Jayatilleke, 2020). Though pre-trained neural word embeddings are widely used, they assume that a word's meaning is relatively stable and does not change across different sentences. Hence these word embedding are not context-specific at the level of different uses of the same word. We used both TF-IDF and GloVe as context-free representations of language in our study to compare the outcomes against context-specific representations introduced below (see Sections 3.2.1, 3.2.2 for details).

Recent work such as ELMo (Peters et al., 2018), BERT (Devlin et al., 2019), and OpenAI GPT (Radford et al., 2018) use fine-tuning methods to further improve the pre-trained word embeddings. Instead of directly using fixed pre-trained neural word embeddings (as in the case of GloVe), these models fine-tune the pre-trained models on downstream tasks and target data to achieve context-dependent word embeddings. For example, the pre-training stage of BERT is typically task-agnostic and the models cannot always capture the domain-specific language patterns well. To improve the pre-trained language models for specific domains, some studies extend BERT on specialty corpora to generate a domain-specific BERT, such as BioBERT (Lee et al., 2020) for biomedical text, SciBERT (Beltagy et al., 2019) for scientific text, ClinicalBERT (Huang et al., 2019) for clinical text. Similar to these studies, we extended BERT with a large interview answer corpus of over 3 million answers (over 330 million words) collected from online candidate interviews. The resulting InterviewBERT contains the general language knowledge already encoded in the initial BERT with the addition of job interview specific knowledge learnt from interview answers. We introduce the details of using InterviewBERT for personality prediction in Section 3.2.3. Many NLP tasks achieve state-of-the-art performance with BERT based methods, including text-based personality predictions using social media text (Christian et al., 2021; Lucky and Suhartono, 2021). Our work remains novel in the context of predicting personality from interview responses using an extended version of the BERT model trained on a very large corpus of interview responses.

### 2.4. BERT and Self-Attention

**Figure 2:**
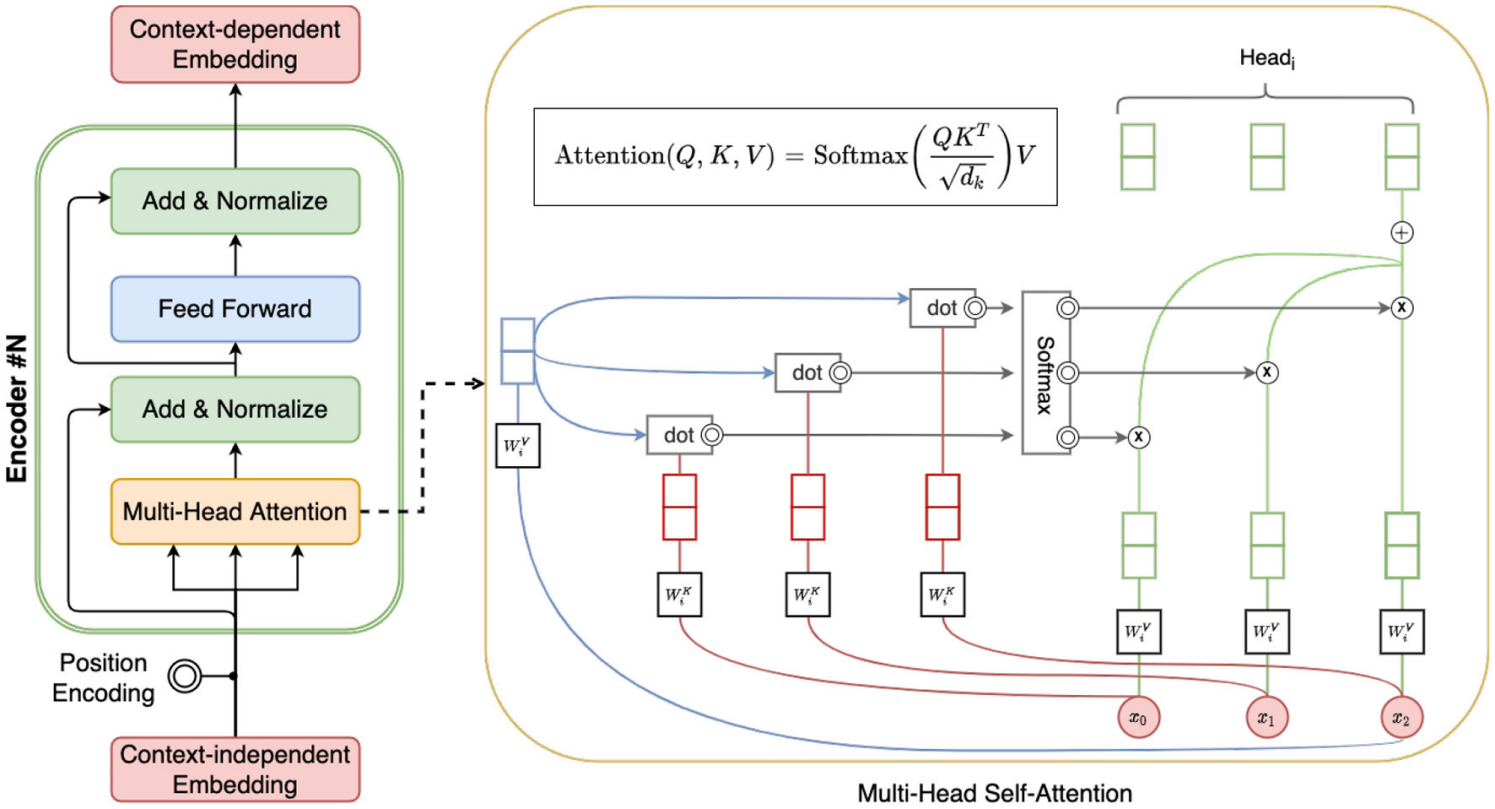
Illustration of BERT and self-attention.

Figure 2 illustrates the overall architecture of BERT, which is a stack of six layers, and each layer has a multi-head self-attention layer and a fully connected feed-forward network. The first token of every input sentence is a special token identified as *[CLS]*. The final hidden state of this token within the BERT model is typically used as the aggregated context-aware representation for the input sentence.

During pre-training, BERT is trained on unlabeled data over different pre-training tasks, including: (1) *predicting the original vocabulary of a randomly masked word in input based only on its context*, and (2) *whether a given sentence is the next sentence of a input sentence*. During this fine-tuning, the BERT model is first initialized based on the general corpus, and then fine-tuned using training data from the downstream tasks. In the case of personality inference, each personality trait has a separate fine-tuned model. We'll introduce the detail of pre-training and fine-tuning of InterviewBERT in Section 3.2.3.

The multi-head self-attention in BERT is illustrated in the right side of Figure 2. Attention is a mechanism to find the words of importance for a given query word in a sentence and multi-head attentions combine the knowledge explored by multiple heads instead of using one. Mathematically, it repeats the self-attention computations multiple times in parallel, and each of them computes attentions based on different aspects of the meanings of each word. With this multi-head self-attention mechanism and the learned context-specific representations of the answer (i.e., *[CLS]*), we can interpret the relationship between the word usage in answers and the predicted personality scores. We show how this ability in BERT can be used to provide better explainability to the personality predictions made by InterviewBERT.

## 3. Materials and Methods

In this section, we discuss the dataset, algorithms and experimental methodology used in achieving the two key aims of this study, namely, training of InterviewBERT and training of individual trait inference models using InterviewBERT. While a lengthy technical discussion of InterviewBERT is out of the scope of this paper, we provide a brief overview in Section 3.2.3. We then demonstrate the use of both context-free (TF-IDF and GloVe) and context-specific (InterviewBERT) representations in building regression models to predict personality traits and compare their accuracy. Given that a candidate typically answers multiple questions in an interview, we explore different ways of aggregating the multiple answers in order to achieve the highest accuracy in the regression task.

### 3.1. Dataset Construction

Our training data comes from the Sapia^1^ FirstInterview™ product, which is an online chat-based interview platform where candidates answer 5–7 open-ended interview questions related to past behavior and situational judgement. The larger data set used to build InterviewBERT included 3,030,018 individual interview question responses from 505,013 candidates. Following are some examples of the open ended questions answered by the candidates.

- *Tell us about a problem you solved in a unique or unusual way. What was the outcome?*
- *Describe a time when you missed a deadline or personal commitment. How did that make you feel?*
- *Give an example of a time you have gone over and above to achieve something. Why was it important for you to achieve this?*
- *Tell us about a time when you have rolled up your sleeves to help out your team or someone else*.

The 5–7 questions in each interview were selected based on the requirements of the role (e.g., retail assistant, sales, call center agent, engineer etc.) and the values sought by the employer. It's important to note that questions are rotated regularly to address gaming risk and plagiarized answers are flagged. On average candidates wrote 110 words per question and were encouraged to write at least 50 words per answer.

Following are two example answers to the question *Tell us about a time when you have rolled up your sleeves to help out your team or someone else?*

- *As captain of my football team I always had to aid my team week in and week out. I had to communicate with the team to ensure everyone was happy in their positions and to ensure our cohesion was at a perfect level to ensure top performance. I would advise each player on one thing they can improve on for the next game and one thing they did particularly well on during the game. Through this method our team was highly successful and was always developing. I thoroughly enjoy working in a team because I love communicating with new people and learning from others*.
- *Whilst working as a tutor whenever another member of staff was sick or unable to come to work that day I was always happy to share out their work load and take on more children than usual for that session. During exam periods we were often spread thin but I was happy to do some extra marking, work a little later and come earlier to help set up the tables and chairs*.

A subset of the candidates (*N* = 58,000) also self-rated themselves on a HEXACO-based personality inventory that provided us with the ground truth to train individual HEXACO trait inference models. It is important to note that not all 58,000 candidates were presented with self-rating items for all six traits due to the strain on candidates to answer both open ended text questions and a further set of close to 50 self-rating questions. Instead, candidates were presented with inventory items to cover at least two traits and a maximum of six. Table 1 shows the number of candidates who answered self-rating items for each of the HEXACO traits and other important statistics.

**Table 1:** Statistics of the dataset used in experiments. Std. is in short for “*standard deviation*” and Ave. is in short for “*average*.” Gender information was not available for all participants.

	**H**	**E**	**X**	**A**	**C**	**O**
Participants	8,317	13,831	23,293	15,683	12,524	15,995
Female %	36	49	49	45	34	53
Male %	41	38	51	55	42	47
Ave. trait score	4.23	2.87	3.79	3.89	4.31	3.30
Std. trait score	0.56	0.47	0.54	0.48	0.43	0.49
Female—Ave. trait score	4.28	2.90	3.74	3.91	4.37	3.25
Male—Ave. trait score	4.18	2.83	3.85	3.88	4.32	3.35
Ave. word length	87.25	100.68	84.95	80.98	83.77	95.75
Std. word length	39.30	56.74	33.74	28.17	35.01	52.63
Female—Ave. word length	109.56	137.74	103.06	97.68	102.47	126.94
Male—Ave. word length	107.74	126.35	101.15	95.71	99.80	113.33

In the model training process for each trait, 80% of the data was used for training, 10% as the development data set for selecting the hyperparameters, and the remaining 10% of the data to validate the accuracy of the trained models. Answers with length less than 50 words were excluded from the training, development and testing data sets as candidate were instructed to answer questions with more than 50 words to provide enough context. More than 70% of candidates provided their gender information and female candidates tended to write longer answers than males on average with respect to the word count.

### 3.2. Answer Representations

We evaluated two commonly used open-vocabulary approaches for text representation, namely, TF-IDF + LDA and GloVe word embedding to compare the outcomes with the proposed InterviewBERT approach. Here we briefly describe how each approach was implemented in the experiment and details of the IntervewBERT development.

#### 3.2.1. TF-IDF and LDA

In this approach, we first remove special characters, numbers and stop words from answers. Then each answer is converted to lowercase and lemmatized before being tokenized. These are typical pre-processing steps in NLP for a context-free representation of textual data. Subsequently, 2,000-dimensional vector representations $v_{i}^{tfidf}$ are formed based on the term frequency-inverse document frequency (TF-IDF) scheme using the 2,000 most common unigrams, bigrams, and trigrams. In the TF-IDF scheme, the value for an answer-term combination increases with the number of times the term is used in the response while offsetting for the overall usage of the term in the whole training dataset. We implemented TF-IDF using sklearn^2^ package.

We also used the Latent Dirichlet Allocation (LDA) (Blei et al., 2003) to derive 100 topics from the answer. LDA assumes the existence of latent topics in a given set of documents and tries to probabilistically uncover these topics. Once uncovered, an answer can be represented with a 100-dimensional vector $v_{i}^{lda}$. We use the Gensim software package^3^ for topic modeling. The combined use of TF-IDF with LDA was shown by Jayaratne and Jayatilleke (2020) to produce the best accuracy in predicting personality from a similar dataset related to interview responses.

We used the same approach to obtain the final answer representation ***V***_***i***_ by concatenating the representation based on terms $v_{i}^{tfidf}$ and the answer representation based on topics $v_{i}^{lda}$ for answer *a*_*i*_:


(1) $$\begin{array}{r} V_{i}=v_{i}^{lda}⊕v_{i}^{tfidf} \end{array}$$

where ⊕ is the concatenating operation and ***V***_***i***_ is a 2100-dimensional vector.

#### 3.2.2. GloVe

GloVe model uses the co-occurrence probabilities of words within a text corpus in order to embed them in meaningful vectors. It first collects word co-occurrence statistics in the form of a word co-occurrence matrix *X*. Element *X*_*ij*_ represents how often the main word *i* appears in the context of word *j* by scanning the corpus with a fixed window size for the main word *i*. Then it learns vectors by doing dimensional reduction on the co-occurrence counts matrix. In this paper, we use the GloVe embeddings that are pre-trained on Common Crawl^4^. The pre-trained model contains 840B tokens with each token represented as a 300-dimensional vector.

We first tokenize answers based on whitespace, newline characters, and punctuation as delimiters. Then, we represent each token as a GloVe embedding $v_{i_{j}}^{glove}$ using torchtext's glove embedding tool^5^. All the out-of-vocabulary tokens are represented as the same vector of *[UNK]*. To get the final answer representation ***V***_***i***_, we averaged across all token representations:


(2) $$\begin{array}{c} V_{i}=\frac{1}{m}\sum_{j=1}^{m}v_{i_{j}}^{glove} \end{array}$$

where *m* is the number of tokens in the answer.

#### 3.2.3. InterviewBERT

To improve the pre-trained BERT language model for interview language understanding, we first extended it with a large interview answer corpus of over 3 million answers. Each answer is first tokenized using WordPiece tokenizer^6^ that also adds a special token [*CLS*] to the start of each answer to enable an answer level representation. InterviewBERT is then pre-trained using the same tasks detailed in Section 2.4. The training process updates the word embeddings based on interview context, while not losing the prior knowledge in general domains. That is, after the pre-training, InterviewBERT contains the general language knowledge already encoded in the initial BERT with the addition of job interview specific knowledge learnt from interview answers.

**Figure 3:**
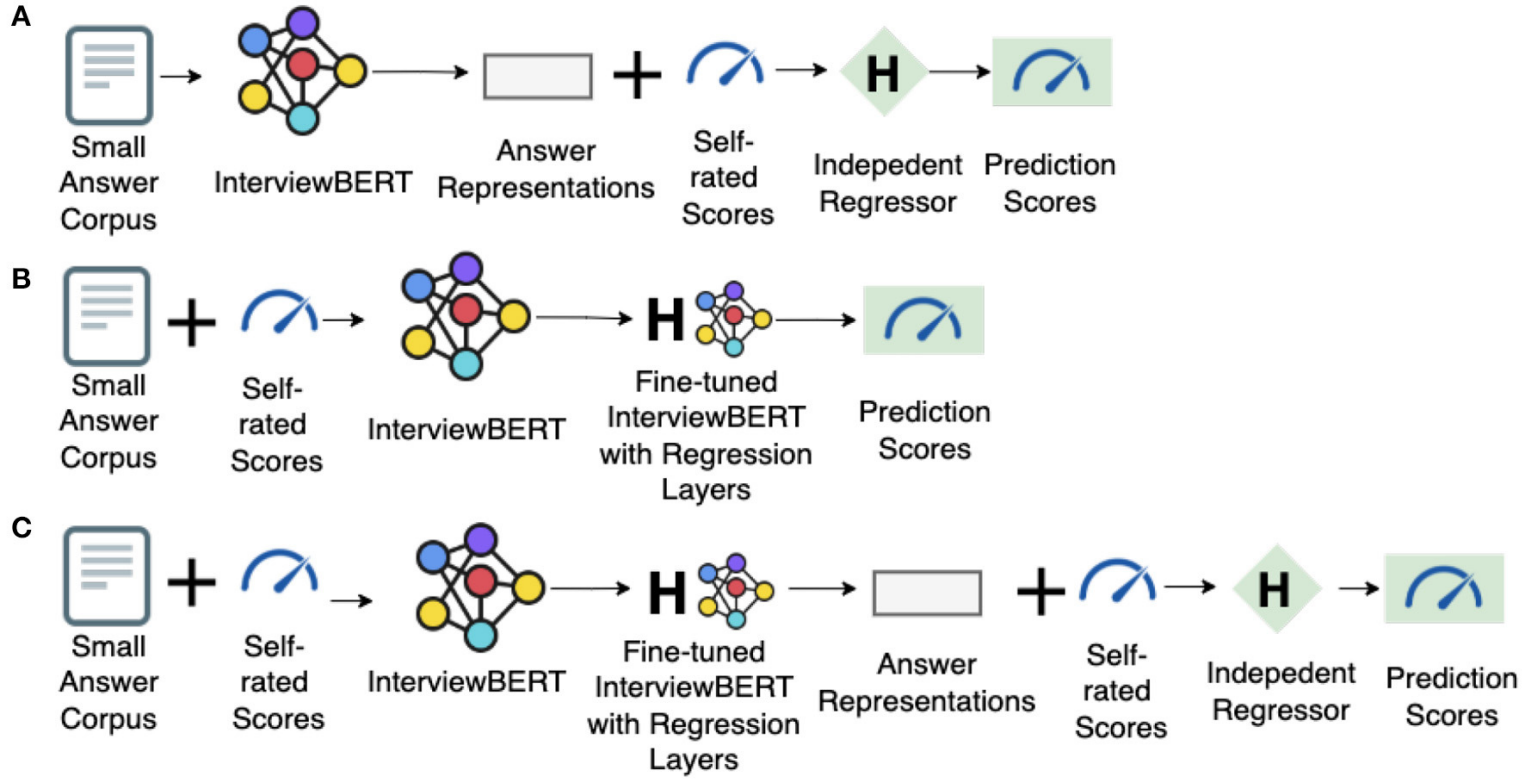
Illustration of different ways of using InterviewBERT. **(A)** Train an independent regressor without fine-tuning. **(B)** Fine-tune InterviewBERT. **(C)** Hybrid approach.

With pre-trained InterviewBERT, we can either fetch answer representations by passing each answer through its encoder and then training a personality predictor based on those representations (i.e., train an independent regressor, as shown in Figure 3A), or fine-tune the pre-trained model itself for personality prediction task (i.e., add a regression layer to InterviewBERT itself, as shown in Figure 3B). A hybrid approach is to get contextualized answer embeddings after fine-tuning InterviewBERT for a regression task and then training a personality predictor separately (as shown in Figure 3C). The advantage of the hybrid approach is that the representation of the same answer could be optimized individually for each regression task (e.g., predicting the trait Extraversion vs. Agreeableness) to obtain better results for the given task than using a generic representation. To make a fair comparison with other methods based on context-free representations, in which the personality predictors are separately trained after obtaining answer representations, we used the hybrid approach in building the InterviewBERT based models.

In order to obtain task and context specific representations we explored two approaches; (a) fine-tuning the model using the learned context-specific [*CLS*] representations (InterviewBERT-CLS) or (b) average all the learned context-specific word embeddings in an answer (InterviewBERT-AVE) as the answer representation. We briefly describe each method below and then report the performance of each approach in Section 4.

**InterviewBERT-CLS** we first input an answer to the encoder of InterviewBERT and get the last hidden representation $v_{i_{cls}}^{T}\in R^{d}$ for *[CLS]* as the answer representation, where *d* = 768. We then passed it through a regression layer to get the personality score $y_{i}^{T}\in R$. The model is fine-tuned by minimizing the mean squared error loss *L* between prediction scores $y_{i}^{T}$ and the ground truth scores ${ŷ}_{i}^{T}$ for trait *T*:


(3) $$\begin{array}{c} L^{T}=\frac{1}{X^{T}}\sum_{i=1}^{X^{T}}{\left(y_{i}^{T}-{\hat{y}}_{i}^{T}\right)}^{2} \end{array}$$

where X is the total number of answers to fine-tune the models for trait *T*.

With the fine-tuned model, we can get context-specific representations $V_{i}^{T}$ by passing answers through the InterviewBERT encoder:


(4) $$\begin{array}{r} V_{i}^{T}=v_{i_{cls}}^{T} \end{array}$$

**InterviewBERT-AVE** instead of using an aggregated answer representation as above, word level representations are averaged to get an answer level representation. We obtained all the hidden word representations from the model except for the [*CLS*] token from the last layer, and then averaged across all word representations to obtain the answer representation $V_{i}^{T}\in R^{d}$. Then, similar to [*CLS*] based representation, we pass $V_{i}^{T}$ through a regression layer to get personality score $y_{i}^{T}$. The model is fine-tuned by minimizing the mean squared error loss *L* between prediction scores and the ground truth scores.

With the fine-tuned model, we can get a context-specific answer representation $V_{i}^{T}$ by passing the answer through the InterviewBERT encoder, and averaging the output word embeddings:


(5) $$\begin{array}{c} V_{i}^{T}=\frac{1}{m-1}\sum_{j=2}^{m}v_{i_{j}}^{T} \end{array}$$

where *m* is the number of tokens in answer *a*_*i*_.

The models are implemented using the Huggingface transformers package^7^ and optimized on Nvidia Tesla T4 using the Adam optimizer (Kingma and Ba, 2014) with β_1_ = 0.9, β_2_ = 0.999, ϵ = 1*e*−6, weight decay tuned among [0.001, *0.01*]. The learning rate, which is warmed up over the first 500 steps, is tuned between [*1e-4*, 1e-3], and then linearly decayed. The model is trained with a dropout, which is tuned between [*0.1*, 0.2], on all layers and attention weights to avoid overfitting. The batch size is tuned between [*8*, 16] and the maximum sequence length is tuned between [256, *512*]. We use the development datasets to select the best hyperparameters and the optimal hyperparameters are highlighted above in *italics*. The model is trained for a maximum of 5 epochs with evaluation for every 2,000 steps. Early stopping is set once convergence is determined, i.e., when the loss *L* on the development set does not decrease after 10,000 steps.

### 3.3. Personality Inference

Once an answer representation is obtained using the context-free and context-specific methods discussed above, the inference task involves building a regressor for each HEXACO trait using the text representation as the independent variable and the self-rating score as the dependent (target) variable. We used the Random Forest algorithm (Breiman, 2001) implemented using sklearn^8^ to train regression models for each trait with a maximum tree depth set to 50 and the number of trees in the forest set to 100. Given each participant responded to 5–7 interview questions but only had a single trait score from the self-report items, two methods were explored to aggregate the answer representations in building the regression model. One method was to train a regression model using each individual answer representation to predict the trait score of a participant and then average scores across answers to get the final individual trait score. Second method was to average all answer representations for a candidate and use the averaged answer representation to train a regression model to predict the trait score. We found that method one provided higher accuracy than method two and hence report results from the first method in Section 4.

## 4. Results

We evaluated the trained models on the 10% of the data set left out for testing using the Pearson correlation coefficient, *r*, between the ground truth personality scores, ŷ, and the predicted personality scores, *p*.

Table 2 presents the performance of the models trained on different answer representation methods. All representation methods produced predictive models with varying levels of positive correlations (*p* < 0.001) for all six HEXACO traits. This demonstrates that language used in responding to interview questions are predictive of one's personality. Further the higher average correlations in context-specific InterviewBERT models over the context-free approaches highlight the superiority of InterviewBERT.

**Table 2:** Correlation coefficient *r* of different methods which aggregate the scores after prediction.

**Methods**		**H**	**E**	**X**	**A**	**C**	**O**	**Ave. *r***
Context-free	TF-IDF + LDA	0.31	0.28	0.41	0.23	0.38	0.39	0.333
	GloVe	0.34	**0.30**	0.41	0.24	0.38	0.39	0.343
Context-specific	InterviewBERT-AVE	**0.37**	**0.30**	**0.44**	**0.28**	0.40	**0.45**	**0.373**
	InterviewBERT-CLS	**0.37**	**0.30**	**0.44**	**0.28**	**0.41**	0.44	**0.373**

*All correlations are significant with p < 0.001. Bold numbers indicate the best correlation for each trait*.

Table 3 presents the correlation between answer word length and the predicted trait scores of different models. This demonstrates that the answer length to interview questions have a weaker correlation with one's personality compared with language use.

**Table 3:** Correlation coefficient *r* between answer length and trait scores predicted by different methods.

**Methods**	**H**	**E**	**X**	**A**	**C**	**O**	**Ave. |*r*|**
Ground truth	−0.04	0.02	0.08	0.01	0.08	−0.04	0.045
TF-IDF + LDA	−0.07	0.17	0.10	0.01	0.10	−0.13	0.096
GloVe	-0.13	0.15	0.07	0.01	0.07	−0.12	0.092
InterviewBERT-AVE	−0.06	0.11	0.16	0.04	0.11	−0.02	0.083
InterviewBERT-CLS	−0.05	0.13	0.15	0.06	0.12	−0.01	0.087

*All correlations are significant with p < 0.001*.

Table 4 presents the inter-correlations between the personality scores inferred using the InterviewBERT-CLS model on an independent group of *N* = 11,433 candidates. This demonstrates that there are strong inter-correlations (|*r*|>0.20) between some personality traits, and these correlations are consistent with previous findings in literature (Ashton and Lee, 2009; Lee and Ashton, 2018; Moshagen et al., 2019; Skimina et al., 2020).

**Table 4:** Intercorrelations between personality scores inferred using InterviewBERT on an independent group of 11,433 candidates.

	**H**	**E**	**X**	**A**	**C**	**O**
Honesty-humility (H)	1.00	–	–	–	–	–
Emotionality (E)	0.18	1.00	–	–	–	–
Extraversion (X)	−0.18	−0.12	1.00	–	–	–
Agreeableness (A)	0.31	0.14	0.05	1.00	–	–
Conscientiousness (C)	0.29	0.20	0.24	0.19	1.00	–
Openness (O)	−0.10	0.11	0.43	−0.11	0.33	1.00

**Figure 4:**
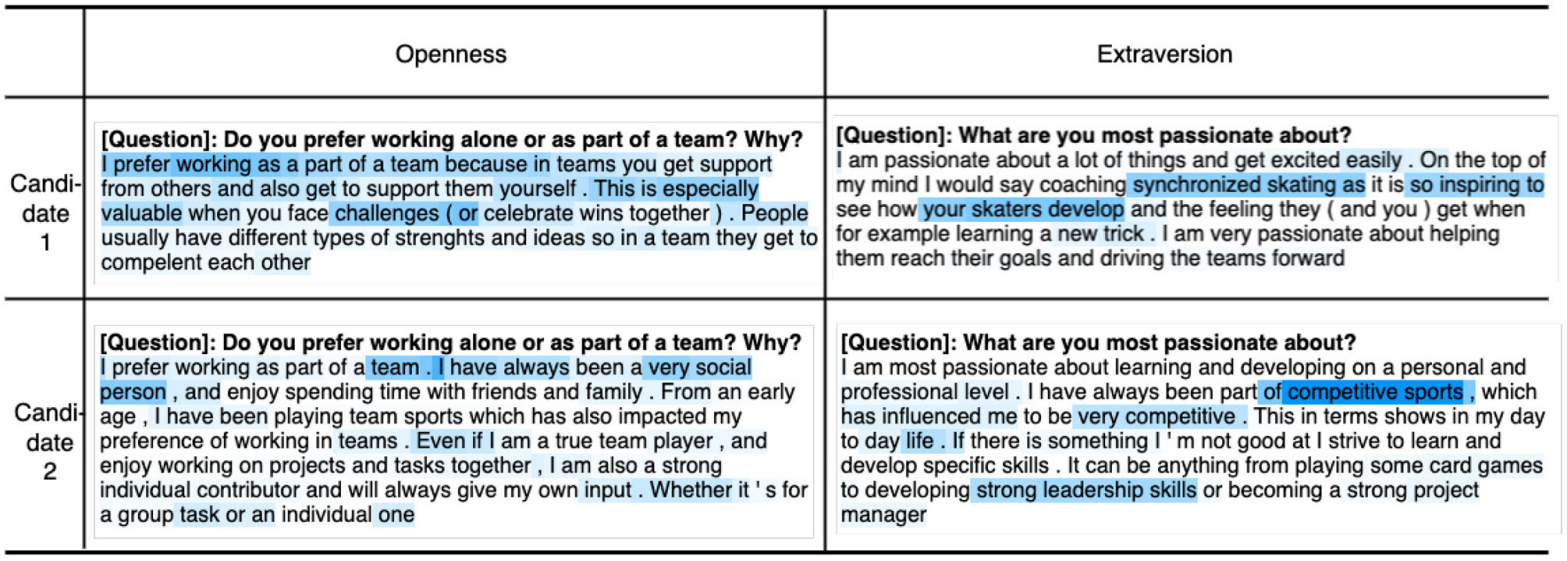
Visualization of attentions from InterveiwBERT along with corresponding tokens for openness (O) and extraversion (X). Tokens with higher attention are in darker color.

Figure 4 shows an example of four attention heatmaps from InterviewBERT for answers from two candidates with corresponding tokens related to Openness (O) and extraversion (X); tokens with higher attention are in darker color. This demonstrates how InterviewBERT can provide us with reasonable language-level explanations of personality inference results allowing further analysis of language patterns related to personality.

Table 5 presents *t*-test results for mean difference between female (*N* = 5,673) and male (*N* = 5,760) on predicted trait scores. These findings of gender differences are similar to that reported by others using standard self-rated item inventories (Feingold, 1994; Ashton and Lee, 2009; Wakabayashi, 2014; Lee and Ashton, 2020; Skimina et al., 2020).

**Table 5:** Mean differences between predicted female (*N* = 5,673) and male (*N* = 5,760) trait scores (*p* < 0.001).

**Traits**	**Diff**	***t***	**Cohen *d***
H	0.04	4.61	0.26
E	0.02	5.71	0.23
X	−0.02	−5.49	−0.16
A	0.02	4.74	0.17
C	0.01	2.0	0.09
O	−0.04	−8.87	−0.31

*Positive differences indicate higher females means*.

## 5. Discussion

The job interview is one of the most widely used assessment tools in the selection process. Personality perception through verbal and non-verbal signals is a common practice used by interviewers in employment interviews. Perceived personality traits of candidates, especially the traits Openness to experience and Conscientiousness, have been found to be positively correlated to interview outcomes (Caldwell and Burger, 1998; Van Dam, 2003). Personality perception is related to the notions of Spontaneous Trait Inference (STI) (Uleman, 1989) and Intentional Trait Inference (ITI) (Uleman, 1999) found in social psychology. Spontaneous trait inferences require little mental effort and are difficult to suppress or modify (closer to being unconscious or automatic), while intentional trait inferences (ITI) require deliberate effort to make a relevant social judgement. In an interview setting the interviewers are expected to get rid of STI, which may unintentionally bring in subjective biases and unavoidably affect the interview results. However, recent research in neuroimaging (Van Duynslaeger et al., 2007; Ma et al., 2011) and social experiments (Ferreira et al., 2012) have shown that STI and ITI often run in synchrony without our awareness. Structured interviews on the other hand attempt to mitigate the impact of interviewer biases by asking the same questions from all candidates with limited interviewer probing and by using a clear scoring rubric to evaluate the candidates based on their responses (Levashina et al., 2013). However, in in-person or video-based structured job interviews where a candidate's appearance or verbal signals are available, it is difficult to avoid interviewers unintentionally forming impressions of candidates influenced by attributes such as race, gender, age, and appearance (Purkiss et al., 2006). Especially when faced with a large number of interviews, human interviewers can hardly infer traits accurately and efficiently.

Our work shows that textual content of interview responses can offer interviewers with rich and deep understanding of a candidate's personality. When used with a structured interview, algorithmic inference of personality from interview responses can help reduce errors in spontaneous trait inference as discussed above. As shown in Table 2 the content and context of answers are strongly correlated with self-reported personality scores. The results also highlight the superiority of context-specific answer representation approaches over context-free approaches by producing the most accurate models. The InterviewBERT based models reached the highest average accuracy of *r* = 0.373 (*p* < 0.001) across the six HEXACO traits while models for Extraversion, Conscientiousness, and Openness exceeded 0.4 correlation, a value typically considered a “correlation upper-limit” for predicting personality with behavior (Meyer et al., 2001; Roberts et al., 2007). Among the different answer representation methods, context-specific methods achieved better results than context-free ones. This is reasonable since context-free methods assume the meaning of a word or a sentence to be relatively stable and unlikely to change across different contexts (see discussion in Section 2.3). On the contrary, context-specific methods are closer to a human in understanding language that consider the context of the words and inter-word correlations in a sentence to better understand the answers. Similar results are also reported on personality prediction using social media data, like tweets and Facebook posts, where context-specific methods (Christian et al., 2021; Lucky and Suhartono, 2021) achieved better performance than context-free methods, such as TF-IDF (Pratama and Sarno, 2015), LDA (Ong et al., 2017), and GloVe (Tandera et al., 2017).

With context-specific methods, traits Extraversion, Conscientiousness, and Openness achieved higher accuracies with correlations exceeding 0.4. These three traits also achieved the highest correlations for context-free methods, albeit only Extraversion exceeding 0.4. Jayaratne and Jayatilleke (2020), also predicting personality from interview responses, report similar results with Conscientiousness and Openness exceeding 0.4 and Extraversion achieving a correlation of 0.34. In a large study using social media data from over 75,000 volunteers, Schwartz et al. (2013) also report their highest correlations for Big5 dimensions Openness, Extraversion, and Conscientiousness at 0.42, 0.38, and 0.35, respectively. On the other hand, we observed that Emotionality and Agreeableness are harder to predict from textual responses with correlations < = 0.3. This is in line with Jayaratne and Jayatilleke (2020) and Schwartz et al. (2013), where they reported their lowest correlations for Agreeableness and Emotionality (Neuroticism in the case of Schwartz et al., 2013 using Big5). The above highlights the different degrees to which language encodes personality signals for different personality traits. Exploring which characteristics of language-use lead to these differences is a useful future direction that is out of scope for this paper.

It is important to highlight here that previous work by Jayaratne and Jayatilleke (2020) using only context-free approaches reached an average correlation of *r* = 0.387 on a similar study with interview responses based personality inference. There are two fundamental differences between this previous study and the current one that we see as improvements, apart from the use of context-specific InterviewBERT. Firstly, the previous study used a concatenated string combining all 5–7 answers per candidate as input to the regressions model while the current study used individual answers to predict personality and then averaged the predicted scores to obtain a final score. Use of individual answers to build InterviewBERT and the proceeding trait prediction models allow the retention of individual answer context compared to combining with other answers. Further the models are less susceptible to the variance in text length due to the varying number of questions in different interviews. Secondly, the TF-IDF + LDA approach used in the previous study lacks the ability to provide explainability as enabled by the InterviewBERT approach.

As shown in Table 3, the average correlations between answer length and personality scores are low (Ave.|*r*| < 0.10). Only Extraversion (X), Conscientiousness (C), and Emotionality (E) have relatively higher correlations with answer length for predicted results (|*r*|>0.10). While further work is required in explaining these higher correlations, some hypotheses can be formed based on the reported characteristics of the traits. For example Extraversion is associated with being sociable and more confident in expressing themselves (McCabe and Fleeson, 2016; Diener and Lucas, 2019), and a long answer may indicate these tendencies. Conscientiousness is associated with striving for accuracy and perfection (McCabe and Fleeson, 2016; Pletzer et al., 2019) and it is reasonable that they tend to answer questions with longer and more elaborate responses. It is interesting to note that the predicted Emotionality (E) scores of all four methods showed correlations of |*r*|>0.10 with the answer length while the correlation with the ground truth remained at 0.02. Further analysis using the explainability features available in InterviewBERT can help explain these correlations by identifying the patterns in language that lead to higher vs. lower scores.

While our context-specific models are trained to predict each personality trait individually, there are inherent inter-correlations among the different personality traits. As shown in Table 4, the HEXACO personality scores inferred by InterviewBERT were weakly correlated overall (Ave. |*r*| ≤ 0.20). However, their are high correlations (*r*>0.20) between Honesty-humility and Agreeableness (H-A), Honesty-humility and Conscientiousness (H-C), Extraversion and Conscientiousness (X-C), Extraversion and Openness (X-O), and Conscientiousness and Openness (C-O). These high correlations have also been reported elsewhere in self-report studies (Ashton and Lee, 2009; Lee and Ashton, 2018; Moshagen et al., 2019; Skimina et al., 2020). (Skimina et al., 2020) report a high inter-correlation for H-A (*r* = 0.44), H-C (*r* = 0.28), X-C (*r* = 0.24), X-O (*r* = 0.22), C-O (*r* = 0.21) based on HEXACO-60 and HEXACO-100 self-rated inventories, which is consistent with the correlations we found based on textual answers to interview questions. Lee and Ashton (2018) report a high H-A correlation in different test groups (0.28 < *r* < 0.42). Moshagen et al. (2019) also found H-A to have the highest correlation and the correlation between X-C to be the second highest. Ashton and Lee (2009) report a high correlation for H-A (*r* = 0.25) and X-O (*r* = 0.26) on a community sample of 734 candidates. These results indicate that our findings of language inferred trait inter-correlations are in-line with other previous studies.

Gender related differences are another aspect where our findings are in line with some of the previous findings (Ashton and Lee, 2009; Lee and Ashton, 2018; Moshagen et al., 2019; Skimina et al., 2020). As shown in Table 5 female candidates show a higher mean difference in Honesty-humility (H) (*d* = 0.26) and Emotionality (E) (*d* = 0.23) compared to males, which is consistent with a study in 48 countries with 347,192 participants (Lee and Ashton, 2020), a study of 522 participants aged 16–75 with 56.3% female (Skimina et al., 2020), and a study of 734 participants with 413 females (Ashton and Lee, 2009). Further, our results show that male candidates on average are higher in Openness to experience (O) than females candidates (*d* = 0.31). While some previous studies have also reported similar results, especially for the Inquisitiveness facet in O with *d* = 0.44 (Skimina et al., 2020) tested on Polish participants, large scale studies such as (Lee and Ashton, 2020) found otherwise. As for Extraversion (X), Agreeableness (A) and Conscientiousness (C), there are no significant differences between female and male candidates (|*d*| < 0.20) and this is consistent with Lee and Ashton (2020), who also tested personality using the HEXACO model.

Explainability is one of the key attributes of ethical use of algorithms together with aspects such as accountability and fairness (Hagendorff, 2020). It addresses the “black-box” problem raised by users of machine learning related to the lack of transparency on how the algorithm works and explaining the outcomes. The ability to see into the “black-box” of the algorithm to get at least a high level understanding of how the outcome is derived increases the user's trust. Using the self-attention mechanism in InterviewBERT (ref. Sections 2.4, 3.2.3) we are able to visualize the attention weights of different words on real interview answers to better examine and understand how various language patterns influence trait outcomes. Figure 4 shows an example of four attention heat-maps for answers from two candidates with corresponding tokens for Openness (O) and Extraversion (X); tokens with higher attention are in darker color. As can be seen, the attention-based methods provide us with reasonable language-level details to analyze the associations learnt by the machine learning models between language patterns and personality. While further work is required in analyzing these associations to discover general patterns (e.g., which words or phrase co-occurrences are more likely to make someone high in Agreeableness), the attention weights in InterviewBERT provide us the data to conduct such a study.

## 6. Conclusion

In this work, we demonstrate how textual content from answers to interview questions related to situational judgement and past behavior can be used to infer personality traits based on the HEXACO model. We extend the Bidirectional Encoder Representations from Transformers (BERT) with a large interview answer corpus of over 3 million answers (over 330 million words) to build InterviewBERT, and use it as the underlying model for personality trait inference from interview responses. The InterviewBERT model is able to better contextualize interview responses based on the interview specific knowledge learnt from the answer corpus in addition to the general language knowledge already encoded in the initial pre-trained BERT. Moreover, we show how “Attention-based” learning approaches in deep neural networks can be used to develop more explainable personality inference models. With regard to gender difference in personality, we show that mean differences in inferred trait scores between male and female groups are similar to those reported by others using standard self-rated item inventories.

Our results show the potential of algorithms to objectively infer a candidate's personality in an explainable manner using only the textual content of interview responses, presenting significant opportunities to remove the subjective biases involved in human interviewer judgement of candidate personality.

For future work, we plan to explore the words and terms discovered through attentions, and analyze how the language usage and the related context are correlated with different personality traits. Since our methodology shows promising results on predicting personality based on individual answers, we are interested in unearthing interview questions that lead to more accurate personality scores from their answers, and explore the effectiveness of different questions. In terms of the underlying algorithms, we are interested in exploring the applicability of other large-scale pre-trained models and different deep regression layers that are jointly fine-tuned with the pre-trained models. Given the inherent inter-correlations among different personality traits, training a multi-task model that could jointly predict different traits is also a direction worth exploring.

## Data Availability Statement

The use of data for this study was permissible under the terms of the Sapia Candidate Privacy Policy to which consent is given by all candidates. Due to legal and privacy restrictions, the authors are not able to make the data publicly available. Requests to access these datasets should be directed to buddhi@sapia.ai.

## Ethics Statement

Ethical review and approval are not required for the study of human participants in accordance with local legislation and institutional requirements. The use of data for this study was permissible under the terms of the Sapia Candidate Privacy Policy to which consent is given by all candidates. All candidate data used for this research has been in de-identified form. No potentially identifiable human images or data are presented in this study.

## Author Contributions

YD, MJ, and BJ contributed to the conception and design of the study. MJ and YD organized the dataset. YD developed the computer code for machine learning model training, performed the statistical analysis, and wrote the first draft of the manuscript. MJ and BJ reviewed the early results of the analysis, provided feedback, and wrote sections of the manuscript. All authors contributed to manuscript revision, read, and approved the submitted version.

## Conflict of Interest

YD, MJ, and BJ were employees of Sapia&Co Pty Ltd. The authors declare that this study received funding from Sapia&Co Pty Ltd. The funder had the following involvement with the study: funded the research and provided the data set on which the research is based.

## Publisher's Note

All claims expressed in this article are solely those of the authors and do not necessarily represent those of their affiliated organizations, or those of the publisher, the editors and the reviewers. Any product that may be evaluated in this article, or claim that may be made by its manufacturer, is not guaranteed or endorsed by the publisher.

## Footnotes

^1^ https://www.sapia.ai/

^2^ https://scikit-learn.org/stable/modules/generated/sklearn.feature_extraction.text.TfidfVectorizer.html

^3^ https://radimrehurek.com/gensim/

^4^ https://nlp.stanford.edu/projects/glove/

^5^ https://torchtext.readthedocs.io/en/latest/vocab.html#glove

^6^ https://huggingface.co/docs/transformers/v4.15.0/en/main_classes/tokenizer

^7^ https://huggingface.co/docs/transformers/v4.15.0/en/index

^8^ https://scikit-learn.org/stable/modules/generated/sklearn.ensemble.RandomForestRegressor.html
